# Study of drowning in fresh and salt water 

**Published:** 2019-07

**Authors:** Mohammadreza Juya, Nasrin Ramezani, Golnaz Peyravi

**Affiliations:** ^*a*^Disaster Risk Management Department, Deputy of Health, Mashhad University of Medical Sciences, Mashhad, Iran.

## Abstract

**Background:**

Drowning occurs when the respiratory tract is blocked by any type of fluid. The factors other than hypoxia can cause death. When water enters the trachea, intense spasm of the larynx causes the trachea to close, so that more water cannot enter the lungs. Sometimes, the spasm may not occur, and the large amount of water entering the lungs lead to drowning.

**Methods:**

This article is a review and analysis of academic texts.

**Results:**

90% of drowning cases occur in freshwaters such as rivers and pools. Drowning in fresh water and entering a large amount of pool or river water into the lungs and stomach is much more dangerous than swallowing a lot of sea water. Swallowing plenty of freshwater leads to quick absorption into blood from the gastrointestinal tract due to a lower osmotic pressure than blood; therefore, it increases blood volume in a short amount of time that results in the loss of red blood cells (hemolysis). Unlike freshwater, saltwater does not induce the above complications because of having the equal osmotic pressure to blood, and it just increases slightly sodium and chlorine causing mild symptoms. For this reason, swimmers are advised that if they swallow a lot of water, they try to remove it from the abdomen, and even if they are in a good condition, they should go to the hospital to control the blood electrolytes, as the symptoms may develop within the next few hours.

**Conclusions:**

The risk of drowning is not merely on busy beaches and can threaten people wherever there is water. Given the possible complications that may occur due to the ingestion of plenty of water after swimming in these places, education and increasing knowledge of people, as well as observing the safety points in this regard is necessary.

**Keywords:**

Saltwater, Drowning, Fresh Water

